# MiR-17-5p regulates cell proliferation and migration by targeting transforming growth factor-β receptor 2 in gastric cancer

**DOI:** 10.18632/oncotarget.8946

**Published:** 2016-04-23

**Authors:** Yanjun Qu, Haiyang Zhang, Jingjing Duan, Rui Liu, Ting Deng, Ming Bai, Dingzhi Huang, Hongli Li, Tao Ning, Le Zhang, Xia Wang, Shaohua Ge, Likun Zhou, Benfu Zhong, Guoguang Ying, Yi Ba

**Affiliations:** ^1^ Tianjin Medical University Cancer Institute and Hospital, National Clinical Research Center for Cancer, Key Laboratory of Cancer Prevention and Therapy, Tianjin, 300060, China

**Keywords:** gastric cancer, TGFBR2, miR-17-5p, cell proliferation, migration

## Abstract

TGFBR2 serves as an initial regulator of the TGF-β signaling pathway, and loss or reduction of its expression leads to uncontrolled cell growth and invasion. TGFBR2 plays a crucial role in the carcinogenesis and malignant process of gastric cancer, but the mechanism remains unclear. In this study, we found that TGFBR2 protein levels were consistently upregulated in gastric cancer tissues, whereas TGFBR2 mRNA levels varied among these tissues, indicating that a post-transcriptional mechanism is involved in the regulation of TGFBR2. MiRNAs are known to regulate gene expression at the post-transcriptional level. Therefore, we performed bioinformatics analyses to search for miRNAs potentially targeting TGFBR2. MiR-17-5p was found to bind to the 3′UTR of TGFBR2 mRNA, and further validation of this specific binding was performed through a reporter assay. An inverse correlation between miR-17-5p and TGFBR2 protein was observed in gastric cancer tissues. Cell studies revealed that miR-17-5p negatively regulated TGFBR2 expression by directly binding to the 3′UTR of TGFBR2 mRNA, thereby promoting cell growth and migration. We also validated the role of TGFBR2 using siRNA and an overexpression plasmid. The results of our study suggest a novel regulatory network in gastric cancer mediated by miR-17-5p and TGFBR2 and may indicate that TGFBR2 could serve as a new therapeutic target in gastric cancer.

## INTRODUCTION

Gastric cancer is the fifth most common malignancy in the world and ranks as the third leading cause of cancer mortality in both men and women, with approximately twice as many deaths in men as in women [1]. The tumorigenesis and progression of gastric cancer involves the activation and deregulation of various signaling pathways, which may represent new therapeutic targets in the future.

MicroRNAs (miRNAs) are endogenous, noncoding, single-stranded RNAs approximately 22 nucleotides in length [2]. MiRNAs regulate gene expression by repressing translation through the ability to bind complementary sequences in 3′-untranslated regions (3′-UTR) of target mRNAs [3, 4]. MiRNAs possess both normal biological functions and function in carcinogenesis and progression [5, 6]. Studies have indicated that more than 30% of human genes are regulated by miRNAs [7–9]. Dysregulation of miRNAs in human cancers may lead to a proto-oncogenic or tumor suppressive role of miRNA in tumorigenesis. Oncogenic miRNAs block the function of certain mRNAs, of tumor suppressors whereas tumor suppressive miRNAs produce an anti-tumor effect [10, 11]. Several studies have reported that miRNAs can regulate several aspects of human gastric cancer pathogenesis, including metastasis, invasion, and self-renewal by targeting the EGR2 [12], MAPK [13] and PI3K/AKT signaling pathways [14]. Therefore, targeting specific miRNAs could be a possible alternative for the treatment of gastric cancer.

Transforming growth factor-β (TGF-β) is a multifunctional cytokine involved in many biological and pathological processes. TGF-β has the ability to act as a tumor suppressor or tumor promoter depending on the cellular environment [15]. In benign epithelia and many early-stage tumors, TGF-β functions as an inducer of growth arrest. However, in advanced tumors, TGF-β promotes tumor progression. There are 3 isoforms of TGF-β (TGF-β-1, TGF-β-2 TGF-β-3) regulating downstream signals [16]. Signal transduction is initiated by the combination of the ligand and the type 1 and type 2 receptor serine/threonine kinases on the cell surface. TGF-β receptor 2 (TGFBR2) subsequently phosphorylates the kinase domain of TGF-β receptor 1 (TGFBR1) [15]. TGFBR1 propagates the signal through the phosphorylation of the downstream proteins SMAD2 and SMAD3, which then polymerize with SMAD4 to form active complexes. The regulatory SMAD complex then translocates to the nucleus, where it acts as a transcription factor regulating target gene expression [17]. TGF-β can also activate other signaling pathways (the so called “non-Smad pathways”), which include the Erk/MAPK pathway [18], the phosphoinositide 3-kinase (PI3K)/AKT pathway [19], c-Src [20] and the mammalian target of rapamycin (mTOR) pathway [21]. These non-Smad pathways work together with or independently from the Smad protein pathway to regulate the function of TGF-β. TGFBR2 is a primary binding protein for all members of the TGF-β family. Loss of TGFBR2 expression enables cancer cells to escape the growth inhibitory effect of TGF-β and may cause loss of cell growth regulation. Several studies have discussed the potential mechanisms of the dysregulation of TGFBR2 [22, 23]. However, other latent mechanisms require further exploration for the clarification of the specific functions of TGFBR2 in carcinogenesis and cancer progression.

MiRNAs and the dysregulation of TGFBR2 are both known to be related to carcinogenesis and progression in gastric cancer. We wondered whether a relationship exists between miRNAs and TGFBR2 regulation and what its biological effects on gastric cancer might be. In this study, bioinformatics prediction provided primary evidence for the specific binding between miR-17-5p and TGFBR2. Subsequently, we provide experimental evidence that TGFBR2 possesses a tumor-suppressive function in gastric cancer and that the protein expression of TGFBR2 was directly suppressed by miR-17-5p in gastric cancer. Our findings provide evidence for the function of miR-17-5p in gastric cancer and help us better understand the modulation of signaling pathways in gastric cancer.

## RESULTS

### TGFBR2 protein levels but not mRNA levels are downregulated in gastric cancer tissues

**Figure 1:**
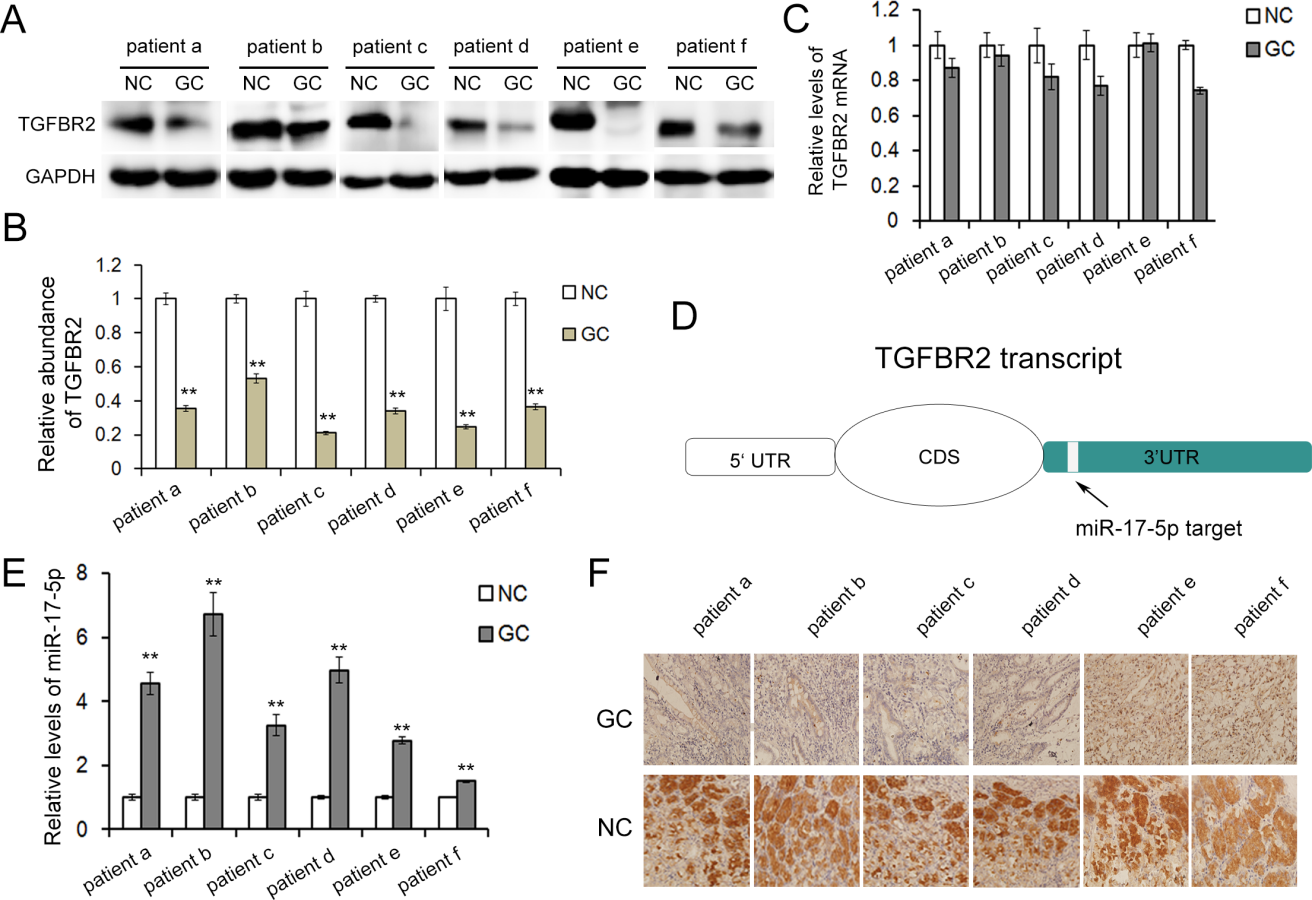
Inverse correlation between TGFBR2 and miR-17-5p in human GC tissues **A.** Western blot analysis of TGFBR2 expression in GC cancer tissues and the paired noncancerous tissues (n=6). **B.** Quantitative analysis of A. **C.** Relative TGFBR2 mRNA levels in GC tissues (n=6). **D.** The predicted binding sites of miR-17-5p in the mRNA of TGFBR2. **E.** Relative levels of miR-17-5p in GC tissues and para-carcinoma tissues (n=6). **F.** Immunohistochemistry of the paraffin-embedded human gastric cancer tissues and paired noncancerous tissues. NC is the paired non-cancerous group of GC. ** indicates p<0.01.

The expression of TGFBR2 in six pairs of human gastric cancer tissues and paired noncancerous tissues was first evaluated by western blotting assay to determine the expression patterns of TGFBR2 in human gastric cancer tissues. Compared with the normal adjacent tissues, the TGFBR2 protein levels were dramatically downregulated in gastric cancer tissues (Figure 1A and 1B). However, there was no significant difference in the mRNA levels between the cancerous and noncancerous tissues (Figure 1C). IHC assays revealed that TGFBR2 was primarily expressed in cancer tissues instead of normal tissues and exhibited a cytoplasmic distribution (Figure 1F).

The protein levels but not the mRNA levels of TGFBR2 were downregulated in gastric cancer, indicating that the expression of TGFBR2 was primarily regulated at a post-transcription level.

### MiR-17-5p is upregulated in human gastric cancer tissues and acts as a potential regulator of TGFBR2

**Figure 2:**
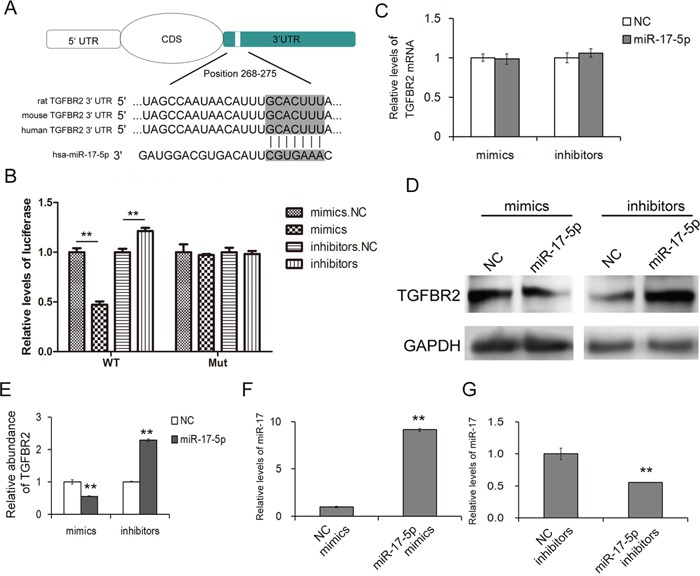
MiR-17-5p regulates TGFBR2 expression in gastric cancer cells **A.** Schematic description of the base-pairing interaction between miR-17-5p and TGFBR2 mRNA. **B.** Direct recognition of TGFBR2 by miR-17-5p. HEK293T cells were co-transfected with firefly luciferase reporters containing either WT or mutant TGFBR2 3′UTR with miR-17-5p mimics and inhibitors. An interaction between miR-17-5p and the target was evident (n=3). **C.** Quantitative RT-PCR analysis of TGFBR2 mRNA levels in SGC-7901 cells treated with miR-17-5p mimics and inhibitors (n=3). **D.** The suppression of TGFBR2 expression by miR-17-5p in SGC7901 cells (n=3). **E.** Quantitative analysis of D (n=3). **F** and **G.** Quantitative RT-PCR analysis of the relative miR-17-5p levels of SGC-7901 treated with miR-17-5p mimics (F) (n=3) and inhibitors (G) (n=3). NC is the corresponding negative control of mimics or inhibitors. ** indicates p<0.01.

MiR-17-5p has been reported to be among the significantly upregulated miRNAs in GC in previous studies [24]. Therefore, the expression level of miR-17-5p was evaluated with real-time PCR in 6 pairs of human gastric cancer tissues and paired matched adjacent noncancerous gastric tissues in this study. The expression level of miR-17-5p was significantly increased in gastric cancer tissues compared with the adjacent noncancerous tissues (Figure 1E). Bioinformatics tools were used to identify the potential miRNAs targeting TGFBR2. As is predicted, miR-17-5p directly binds a region in 3′UTR of TGFBR2 mRNA. The binding site is highly conserved among species (Figure 1D and Figure 2A). Therefore, we selected miR-17-5p for further experiments to verify its specific binding to TGFBR2 and thus the regulation of its downstream signaling pathways.

### Validation of TGFBR2 as a direct target of miR-17-5p

MiRNA-mediated suppression of mRNA transcription is thought to be one of the important types of post-transcriptional regulation. Based the prediction of the bioinformatics tools, a luciferase assay was performed in HEK-293T cells to evaluate the direct interaction between miR-17-5p and TGFBR2. The relative luciferase activity was clearly inhibited when miR-17-5p mimics were co-transfected with the luciferase reporters containing the predicted binding region of the wild type 3′UTR of TGFBR2. However, the interaction was lost when a plasmid with a mutated sequence was used instead. Furthermore, the co-transfection of miR-17-5p inhibitors and the plasmid with the wild TGFBR2 3′UTR resulted in a relative increase in the luciferase signal (Figure 2B).

The same biological effect of miR-17-5p on TGFBR2 was evaluated in SGC-7901 cells. The expression of miR-17-5p was confirmed by qRT-PCR (Figure 2F and 2G). Overexpression of miR-17-5p led to a sharp reduction of TGFBR2, whereas the inhibition of miR-17-5p slightly enhanced the expression of TGFBR2 (Figure 2D and 2E). Furthermore, miR-17-5p did not affect the mRNA levels of TGFBR2 in SGC-7901 cells (Figure 2C).

The results demonstrated that miR-17-5p regulated TGFBR2 by directly binding the specific region of the 3′-UTR of TGFBR2.

### Upregulation of miR-17-5p promotes the proliferation and migration of SGC-7901 cells

**Figure 3:**
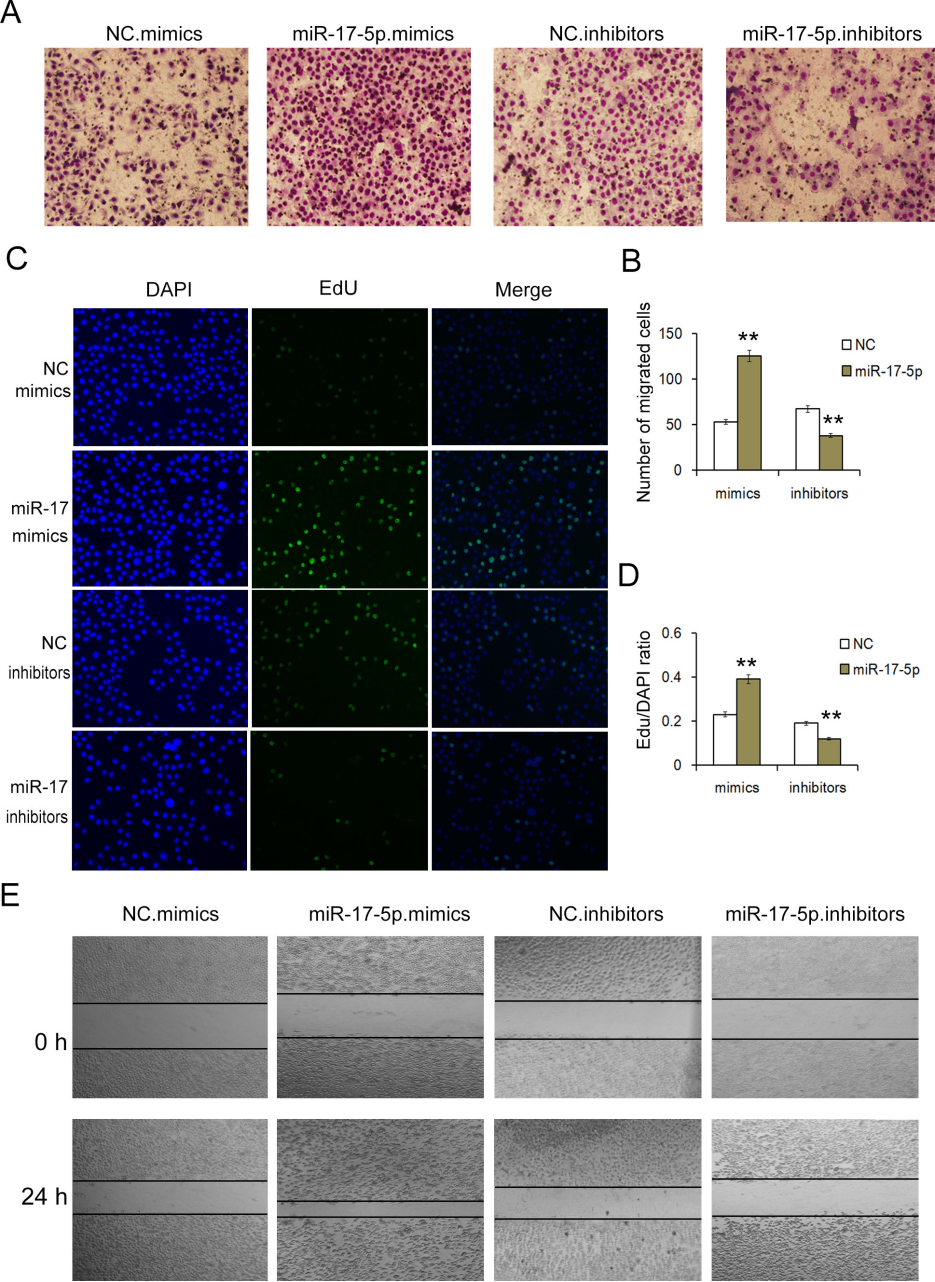
MiR-17-5p regulates the proliferation and migration of GC cells **A.** Transwell assays demonstrate that miR-17-5p promotes the migration of GC cells. Overexpression of miR-17-5p promotes cell migration, whereas low levels of miR-17-5p suppress GC cell migration in GC (n=3). **B.** Quantitative analysis of A (n=3). **C.** EdU assays demonstrate that miR-17-5p promotes the proliferation of GC cells. Overexpression of miR-17-5p promotes cell proliferation, whereas low levels of miR-17-5p suppress GC cell proliferation (n=3). **D.** Quantification of C (n=3). **E.** Validation of miR-17-5p-mediated cell migration by the scraping line method (n=3). NC is the corresponding negative control of mimics or inhibitors. ** indicates p<0.01.

We next examined the biological effects of miR-17-5p and TGFBR2 on gastric cancer cells. The Cell-Light EdU DNA cell kit was used to measure the cell proliferation rates of SGC-7901 cells. As expected, the overexpression of miR-17-5p resulted in increased cell proliferation, whereas knock-down of miR-17-5p led to the inhibition of cell proliferation (Figure 3C and 3D).

A wound healing assay was used to examine the migration ability of SGC-7901 cells. Twenty-four hours after transfection, a single scratch wound was created in each well, and the wound closure was monitored. The upregulation of miR-17-5p significantly promoted cell migration (Figure 3E).

We also conducted transwell assays to evaluate the migration capacity of transfected cells. We found that compared with the control cells, the upregulation of miR-17-5p expression promoted the migration of gastric cancer cells, whereas the downregulation of miR-17-5p expression inhibited migration (Figure 3A and 3B).

The above results suggest that miR-17-5p acts as an onco-miR in human gastric cancer and that the upregulation of miR-17-5p expression promotes cell growth and migration ability in SGC-7901 cells.

### Effects of TGFBR2 on SGC-7901 cells proliferation and migration

**Figure 4:**
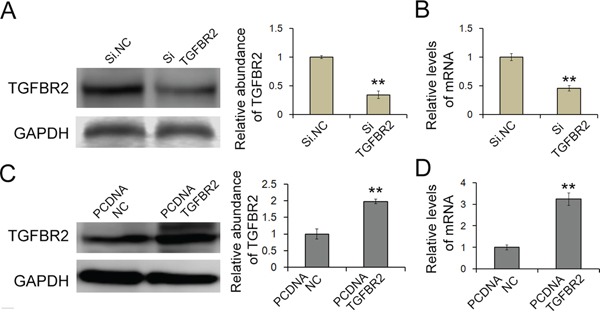
Knock-down of TGFBR2 in SGC7901 cells **A** and **B.** Silencing of TGFBR2 expression by siRNA. SGC7901 cells were transfected with TGFBR2 siRNA, and the protein and mRNA levels were measured (n=3). **C** and **D.** Overexpression of TGFBR2 using an overexpression plasmid; the protein and mRNA levels were measured (n=3). Si.NC is the negative control of siRNA of TGFBR2. PCDNA.NC is the negative control of TGFBR2 overexpression plasmid. ** indicates p<0.01.

We next investigated the effects of the overexpression and silence of the TGFBR2 on cell proliferation and migration. The siRNA sequence targeting human TGFBR2 cDNA was used to knock down the expression of TGFBR2, and a plasmid expressing the ORF of TGFBR2 was designed to overexpress TGFBR2. The silencing and overexpression efficiency was assessed by RT-qPCR for RNA and by western blotting for protein. As is shown in the figures, both the mRNA and protein levels of TGFBR2 were markedly inhibited by siRNA and were augmented by the overexpression plasmid (Figure 4A, 4B, 4C and 4D).

**Figure 5:**
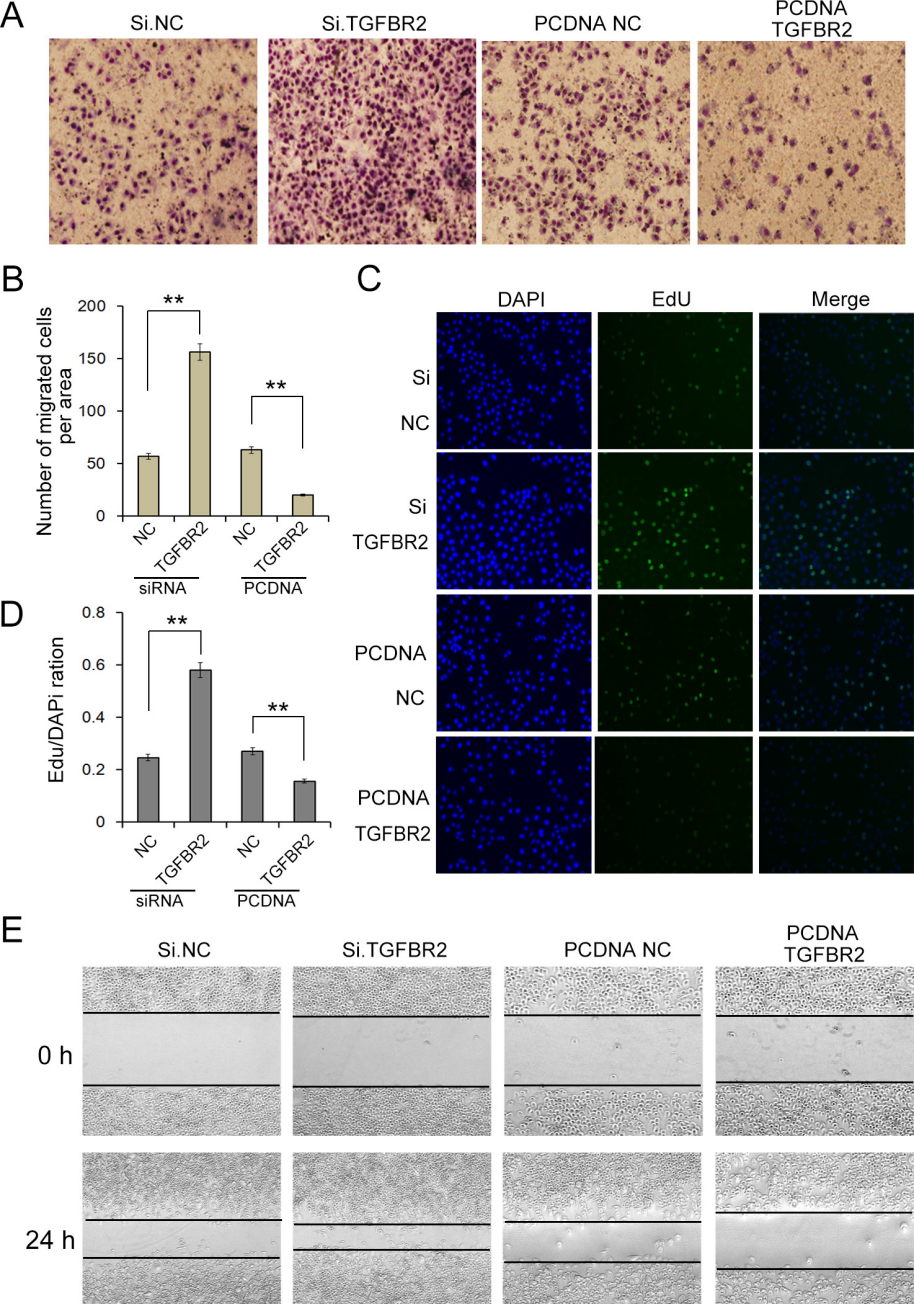
Overexpression or silence of TGFBR regulates cell proliferation and migration in SGC-7901 cells **A.** Transwell assays demonstrate that knock-down of TGFBR2 strongly enhances cell migration in GC cells, whereas overexpression of TGFBR2 inhibits cell migration (n=3). **B.** Quantitative analysis of A (n=3). **C.** Silencing of TGFBR2 clearly promotes the proliferation of SGC7901 cells, whereas overexpression of TGFBR2 inhibits cell proliferation (n=3). **D.** Quantitative analysis of C. **E.** Validation of miR-17-5p-mediated cell migration by the scraping line method (n=3). Si.NC is the negative control of siRNA of TGFBR2. PCDNA.NC is the negative control of TGFBR2 overexpression plasmid. ** indicates p<0.01.

SGC-7901 cells in which TGFBR2 was knocked down exhibited a significantly higher rate of proliferation and increased migration, whereas the cells overexpressing TGFBR2 exhibited a significantly lower rate of proliferation and decreased migration (Figure 5A, 5B, 5C, 5D and 5E). Thus, TGFBR2 exhibits a cancer-suppressive function in gastric cancer, the dysregulation of which may increase the proliferation and migration of cancer cells.

## DISCUSSION

Identifying the mechanisms underlying carcinogenesis and tumor progression remains a challenge for the current study of cancer. Tumorigenesis and malignant progression is promoted by various genes and proteins, thus leading to uncontrolled cell growth and invasion. Insight into the molecular mechanism underlying carcinogenesis and tumor progression is crucial in the treatment of cancers. Therefore, a better understanding of miRNAs and their target genes may provide new effective therapeutic agents for gastric cancer diagnosis and treatment in the future.

In this study, we detected an inverse expression pattern of miR-17-5p and TGFBR2 in human gastric cancer and paired noncancerous tissues. We verified that TGFBR2 was a direct target of miR-17-5p, consistent with the prediction of bioinformatics tools, and that miR-17-5p could promote the proliferation and migration of gastric cancer cells by negatively regulating the expression of TGFBR2. The cancer-suppressive function of TGFBR2 was further confirmed in gastric cancer cells through the silencing and overexpression of TGFBR2.

MiRNAs are known to modulate gene expression at the post-transcriptional level and has been thought to be a useful diagnostic marker for different cancers and is often related to disorders of protein expression. A number of miRNAs have been reported to be related to tumorigenesis, invasion and metastasis, including miR-21 [25, 26], miR-143 [27–29], miR-27 [30], in gastric cancer. Recent studies also reported that miRNAs can serve as biomarkers for the prediction of prognosis and sensitivity to chemotherapy and radiotherapy in cancers [31–34]. It is well-established that miRNAs are involved in gastric cancer carcinogenesis and may serve as tumor promoters or suppressors. Studies in our group have demonstrated the dysregulation of miRNAs, including miR-17-5p, in gastric cancer. MiR-17-5p is a member of the miR-17-92 miRNA cluster, previously known as oncomiR-1, which contains miR-17-5p, miR-17-3p, miR-20a, miR-19a, miR-19b, miR-92a and miR-18a. Although disorders of miR-17-5p have been reported to be correlated with metastasis and invasion in hepatocellular carcinoma, ovarian cancer, breast cancer, and prostate cancer, the mechanism varied among cancer types and remains largely unknown in gastric cancer. It was demonstrated in our study that miR-17-5p was highly upregulated in gastric cancer tissues and exhibited a cancer-promoting function in gastric cancer.

TGF-β has been known to contribute to tumor-suppressive activity. However, a reduction in TGF-β signaling in tumor cells is often accompanied by increased secretion of the ligand, which can function independently by promoting tumorigenesis and tumor progression through effects on cell proliferation and cell invasion [35]. TGFBR2 is known to act as a tumor suppressor by initiating the TGF-β signaling pathway through recruitment of TGFBR1, thus activating downstream signaling to negatively regulate cell proliferation. Reduced expression or inactivation of TGFBR2 is frequently associated with the loss of sensitivity to the anti-proliferative effects of TGF-β, which is thought to have a strong correlation with carcinogenesis and malignant progression [15, 36]. Furthermore, TGFBR2 is a frequent locus of inactivating mutations, and the mechanism of TGFBR2 dysregulation vary among different cancers. The identification of these mechanisms may provide new therapeutic strategies for cancers. The current study demonstrated a post-transcriptional regulation strategy for TGFBR2 and its negative cancer-promoter function in gastric cancer cells.

To conclude, miR-17-5p promoted cell proliferation and migration by targeting TGFBR2 in gastric cancer. The results of our study depict a novel regulatory network in gastric cancer mediated by miR-17-5p and TGFBR2 and offer potential therapeutic targets in gastric cancer.

## MATERIALS AND METHODS

### Human tissue

Gastric cancer tissues and paired adjacent noncancerous tissues were obtained from patients undergoing surgical procedures at the Tianjin Medical University Cancer Institute and Hospital (Tianjin, China). Both the tumor and noncancerous tissues were histologically confirmed. The pathological type of each cancer tissue was confirmed as adenocarcinoma. Written consent was provided by all the patients, and the Ethics Committee of Tianjin Medical University Cancer Institute and Hospital approved all aspects of this study. Tissues were immediately frozen in liquid nitrogen at the time of surgery and were stored at −80°C.

### Cell line and culture

The human gastric cancer cell line SGC-7901 was purchased from the Shanghai Institute of Cell Biology of the Chinese Academy of Sciences (Shanghai, China). SGC-7901 cells were cultured in a humidified incubator at 37°C with 5% CO_2_ in RPMI-1640 medium (Gibco, USA) containing 10% fetal bovine serum (Gibco, USA) and 1% penicillin/streptomycin (Solarbio, China) in a humidified incubator.

### RNA isolation and quantitative RT-PCR

Total RNA was isolated from the cultured cells and tissues using TRIzol reagent (Invitrogen, USA) according to the manufacturer's protocol. RNA concentrations were determined spectrophotometrically. RNA quality was confirmed using a Nanodrop 1000 spectrophotometer (Thermo Fisher Scientific, USA). First-strand cDNA was synthesized from 1 μg of total RNA by AMV reverse transcriptase (TaKaRa, China). The reaction conditions were as follows: 16°C for 15 min, 42°C for 60 min and 85°C for 5 min. The expression level of miR-17-5p was analyzed by TaqMan miRNA probes (Applied Biosystems, USA). Gene-specific PCR products were assayed continuously using a CFX96 Real-Time PCR system. The PCR was incubated in a 96-well optical plate, initiated by 5-min hold at 95°C, followed by 40 cycles of denaturation at 95°C for 15 s and annealing/extension at 60°C for 1 min. All the reactions were performed in triplicate. U6 snRNA was used as an internal control for miRNA. The mRNA levels of TGFBR2 were normalized to GAPDH. The ΔCt (cycle threshold) method was adopted and applied to calculate the relative quantities of target genes. The relative levels of the target genes were normalized to the control using the equation 2^−ΔCt^, in which ΔCt = Ct _gene_ – Ct _control_. The primer sequences for TGFBR2 and GAPDH were as follows: TGFBR2 sense: 5′-GTAGCTCTGATGAGTGCAATGAC-3′; TGFBR2 antisense: 5′-CAGATATGGCAACTCCCAGTG-3′; GAPDH sense: 5′-AGAAGGCTGGGGCTCATTTG-3′; GAPDH antisense: 5′-AGGGGCCATCCACAGTCTTC-3′.

### Cell transfection

SGC-7901 cells were seeded in a 6-well plate and were transfected using Lipofectamine 2000 (Invitrogen) and Opti-MEM (Gibco, USA) 24 h later according to the manufacturer's instructions. For miRNA overexpression and downregulation, 100 pmol miR-17-5p mimics, inhibitors and negative control were used. For TGFBR2 overexpression and downregulation, the full-length open reading frame (ORF) of human TGFBR2 without the miR-17-5p-responsive 3′-UTR was purchased from FulenGen (Guangzhou, China). An empty plasmid was used as a negative control. The siRNA sequence (5′-GATTCAAGAGTATTCTCACTT-3′) targeting human TGFBR2 was designed and synthesized by Ribobio (Guangzhou, China). A scrambled siRNA (Ribobio) was used as a negative control. The cells were harvested 24 h or 48 h after transfection to isolate total RNA and total cell lysate for q-PCR and western blot analysis, respectively.

### Luciferase reporter assay

The amplified PCR products of human wild type TGFBR2 and a mutant TGFBR2 in which the predicted 3′ UTR miR-17-5p targeting regions were inserted into the pMIR-REPORT plasmid (Ambion, USA). The successful insertion of this sequence was confirmed through DNA sequencing. For luciferase reporter assays, 2 mg of firefly luciferase reporter plasmid, 2 mg of β-galactosidase expression vector (Ambion), and equal amounts (200 pmol) of mimics, inhibitors, or scrambled negative control RNA were transfected into cells. A β-galactosidase expression vector was used as a transfection control. Cells were assayed using a luciferase assay kit (Promega) 24 h after transfection.

### Protein extraction and western blot

Cells and tissues were lysed in RIPA buffer with freshly added protease inhibitor cocktail. Total cell lysates were separated on SDS-PAGE gels and transferred to PVDF membranes (Millipore). Immunoblotting was performed with monoclonal anti-TGFBR2 antibodies (sc-400, Santa Cruz Biotechnology) overnight after blocking with 2% BSA. After incubation with the secondary antibody, the membranes were visualized with an enhanced chemiluminescence system kit (Millipore, USA) according to the manufacturer's protocol. All samples were normalized to GAPDH.

### Cell proliferation assay

Cells seeded in 24-well plates were first transfected with miR-17-5p mimics, inhibitors, TGFBR2 overexpression plasmid, TGFBR2 siRNA and the relevant negative control. Twenty-four hours after transfection, EdU was added to the culture medium at a concentration of 50 μM/ml for 5 h to chase the DNA template, following the instructions of the Cell-Light EdU DNA cell kit (Apollo 488, RiboBio, China). Briefly, after fixation in 4% paraformaldehyde and treatment with 0.5% Triton-X for 15 min, cells were incubated in darkness with Apollo, and nuclei were stained by Hoechst 33342. EdU-labeled cells were counted manually in five fields of view randomly selected from each well, and percentages were calculated.

### Transwell tumor cell migration assay

Twenty-four-well Boyden chambers with 8-μm pore size polycarbonate membrane (Corning, NY) were used to evaluate cell motility. Cells were first transfected with miR-17-5p mimics, inhibitors, TGFBR2 overexpression plasmid, TGFBR2 siRNA and the relevant negative control. Approximately 10^5^ cells were seeded on the upper chamber with 200 μl serum-free medium 24 h after transfection. Approximately 600 μl medium with 10% serum was added to the lower chamber as a chemoattractant. Twenty-four hours after incubation, the membranes were fixed with methanol and stained with a three-step stain set (Thermo, UK). Five visual fields were randomly selected from each membrane. All experiments were performed in triplicate.

### Wound scratch assay

Each group of SGC-7901 cells (transfected with miR-17-5p mimics, inhibitors, TGFBR2 siRNA, overexpression TGFBR2 plasmid and the relevant controls) were seeded in six-well plates. On the following day, when the cells were approximately ≥90%, each well was scraped with a 20 μl pipette tip to create 2 linear regions devoid of cells. Subsequently, the cells in each well were cultured with RPMI-1640 medium (Gibco, USA) containing 2% fetal bovine serum (Gibco, USA) in a humidified incubator. We monitored wound closure at 0 h, 12 h, 18 h, and 24 h after scraping. Five random non-overlapping images of each well were selected and quantitated for statistical analysis.

### Immunohistochemistry assay

Paraffin-embedded specimens of gastric cancer and the paired noncancerous tissues were sectioned and stained with an anti-TGFBR2 monoclonal antibody (sc-400, Santa Cruz Biotechnology) at a 1:50 dilution. The DAB system (Zhongshanjinqiao, China) was used to identify the positive staining. Five random fields were selected for each specimen.

### MiRNA target prediction

MiRNA target prediction and analysis were performed with the algorithms from TargetScan (http://www.targetscan.org/), PicTar (http://pictar.mdc-berlin.de/) and miRanda (http://www.microrna.org/).

### Statistical analysis

The results are presented as the average of at least three experiments, each performed in triplicate, with standard errors. Statistical analyses were performed by an analysis of variance followed by the *t* test using SPSS 20.0. *P* values of 0.05 were considered significant and are indicated with asterisks.
